# Maternal and neonatal outcomes associated with breech presentation in planned community (home and birth center) births in the United States: A prospective observational cohort study

**DOI:** 10.1371/journal.pone.0305587

**Published:** 2024-07-22

**Authors:** Robyn Schafer, Marit L. Bovbjerg, Melissa Cheyney, Julia C. Phillippi

**Affiliations:** 1 Division of Advanced Nursing Practice, School of Nursing, Rutgers University, Newark, NJ, United States of America; 2 Department of Obstetrics, Gynecology, and Reproductive Sciences, Robert Wood Johnson Medical School, Rutgers University, New Brunswick, NJ, United States of America; 3 Epidemiology Program, College of Public Health and Human Sciences, Oregon State University, Corvallis, OR, United States of America; 4 Department of Anthropology, Oregon State University, Corvallis, OR, United States of America; 5 School of Nursing, Vanderbilt University, Nashville, TN, United States of America; Lausanne University Hospital: Centre Hospitalier Universitaire Vaudois (CH), FRANCE

## Abstract

**Objective:**

Investigate maternal and neonatal outcomes associated with breech presentation in planned community births in the United States, including outcomes associated with types of breech presentation (i.e., frank, complete, footling/kneeling)

**Design:**

Secondary analysis of prospective cohort data from a national perinatal data registry (MANA Stats)

**Setting:**

Planned community birth (homes and birth centers), United States

**Sample:**

Individuals with a term, singleton gestation (N = 71,943) planning community birth at labor onset

**Methods:**

Descriptive statistics to calculate associations between types of breech presentation and maternal and neonatal outcomes

**Main outcome measures:**

*Maternal*: intrapartum/postpartum transfer, hospitalization, cesarean, hemorrhage, severe perineal laceration, duration of labor stages and membrane rupture

*Neonatal*: transfer, hospitalization, NICU admission, congenital anomalies, umbilical cord prolapse, birth injury, intrapartum/neonatal death

**Results:**

One percent (n = 695) of individuals experienced breech birth (n = 401, 57.6% vaginally). Most fetuses presented frank breech (57%), with 19% complete, 18% footling/kneeling, and 5% unknown type of breech presentation. Among all breech labors, there were high rates of intrapartum transfer and cesarean birth compared to cephalic presentation (OR 9.0, 95% CI 7.7–10.4 and OR 18.6, 95% CI 15.9–21.7, respectively), with no substantive difference based on parity, planned site of birth, or level of care integration into the health system. For all types of breech presentations, there was increased risk for nearly all assessed neonatal outcomes including hospital transfer, NICU admission, birth injury, and umbilical cord prolapse. Breech presentation was also associated with increased risk of intrapartum/neonatal death (OR 8.5, 95% CI 4.4–16.3), even after congenital anomalies were excluded.

**Conclusions:**

All types of breech presentations in community birth settings are associated with increased risk of adverse neonatal outcomes. These research findings contribute to informed decision-making and reinforce the need for breech training and research and an increase in accessible, high-quality care for planned vaginal breech birth in US hospitals.

## Introduction

There has been a recent increase in breech birth in community settings (homes and birth centers) in the United States [1]. This is despite research demonstrating increased risk of intrapartum or neonatal death (16.8/1000 adjusted odds ratio [aOR] 8.2, 95% CI, 3.7–18.4) [2] in breech community births and consensus obstetric and midwifery recommendations that classify breech presentation as a contraindication to home birth [3, 4]. Since 2000, planned cesarean has been the standard of care for breech presentation, following a landmark large-scale, randomized controlled trial (the Term Breech Trial) [5] and subsequent American College of Obstetricians and Gynecologists (ACOG) committee opinion [6] recommending planned cesarean delivery for all singleton term breech fetuses. However, more recent research has called those recommendations into question [7–12], concluding that although risk of adverse outcomes is higher in planned vaginal breech birth than planned cesarean, the absolute risk is quite low [13–16]. Internationally, support for vaginal breech birth is increasing [17–20], but nearly all breech fetuses (95.5%) in the US are born via cesarean [1, 13, 14, 21]. ACOG committee opinion now recommends that for a term, singleton fetus, planned vaginal breech birth “may be reasonable under hospital-specific protocol guidelines for eligibility and labor management” [22]. However, hospital-based care for planned vaginal breech birth in the US is very difficult to obtain, in part due to a lack of skilled providers and medicolegal concerns [22–24], leading some individuals to seek care in community-based settings (homes and birth centers) [25–27].

Breech presentation affects approximately 3–4% of term pregnancies, and community births currently comprise about 2% of US births [28, 29]. Based on birth certificate data from the National Center for Health Statistics, rates of US community births rose 33.2% from 2019 to 2022, including a 61.7% increase in breech births (n = 423 in 2019, n = 684 in 2022), in tandem with a decrease in hospital births [1]. In 2022, 12.5% (n = 488) of all reported singleton, term (greater than or equal to 37 + 0/7 weeks’ gestation) vaginal breech births in the US occurred in a community birth setting [1]. Research has established that intrapartum and neonatal death rates are higher in breech birth than cephalic births [2], but little is known about neonatal and maternal outcomes associated with breech presentation managed in community birth settings.

Data is also limited about maternal and neonatal outcomes based on type of breech presentation. Breech presentation is classified based on the position of the lower fetal extremities (see **Table 1**). Breech presentation nomenclature has been applied inconsistently in research and clinical practice recommendations, and there is ambiguity about variations of presentation types (such as partial flexion, location of feet alongside or just below the buttocks, or dynamic presentations that change during labor) [5, 15, 18, 30–32]. Alternative nomenclatures have been proposed, but none have gained widespread acceptance [33, 34]. Footling or kneeling breech presentation is generally considered a contraindication to vaginal birth due to increased risk of perinatal morbidity from umbilical cord prolapse or head entrapment leading to hypoxic injury [17–19, 22]. However, there is limited evidence to support this recommendation since, with rare exceptions [30, 35], vaginal breech trials historically have excluded (or not reported data regarding) footling or kneeling presentations [5, 15, 16, 36, 37]. Research that examines potential differences in community birth outcomes associated with type of breech presentation is needed to guide informed decision-making and optimize perinatal outcomes [2]. The purpose of this study was to analyze associations between breech birth and maternal and neonatal outcomes compared to cephalic presentations in planned community births and assess differences in outcomes associated with type of breech presentation.

**Table 1:** Breech presentation nomenclature.

Type	Attitude at hip	Attitude at knee	Position of feet
Frank (or “extended”)	Flexed (both)	Extended (both)	Proximal to the fetal head
Complete (or “flexed”)	Flexed (both)	Flexed (both)	Lack of consensus*
Incomplete^†^	Lack of consensus	Lack of consensus	Lack of consensus
Footling (single or double footling)	Extended (partially or fully, one or both)	Flexed or extended	Presenting below the level of the buttocks
Kneeling (single or double kneeling)	Extended (one or both)	Flexed (one or both)	Below the level of the buttocks and above the level of the knee(s), with one or both knees presenting

Notes

* There is not a consensus definition for position of the fetal feet in a complete presentation, which either (a) cannot be below the fetal buttocks [5] or (b) may be palpable at or just below the buttocks [16, 18].

^†^ The term “incomplete” is inconsistently defined in the literature as either (a) both hips flexed with one knee flexed and one knee extended [18, 30] or (b) one or both hips not completely flexed, regardless of attitude at the knee (in essence, an umbrella term for footling and kneeling presentations) [38, 39].

## Materials and methods

This cohort study used registry data (birth years 2012–2018) from the Midwives Alliance of North America Statistics Project (MANA Stats). MANA Stats includes extensive prenatal, birth, and postpartum data from individuals who received care from midwives in community birth settings in the United States. Individuals are prospectively enrolled in the registry at the onset of care in pregnancy with informed consent, and midwives enter data throughout perinatal care. MANA Stats development, data collection protocols, and evidence of reliability and validity are described elsewhere [40, 41]. Ethical approval was received from Oregon State University’s IRB. All pregnant persons and midwives gave informed consent for research participation.

**Fig 1:**
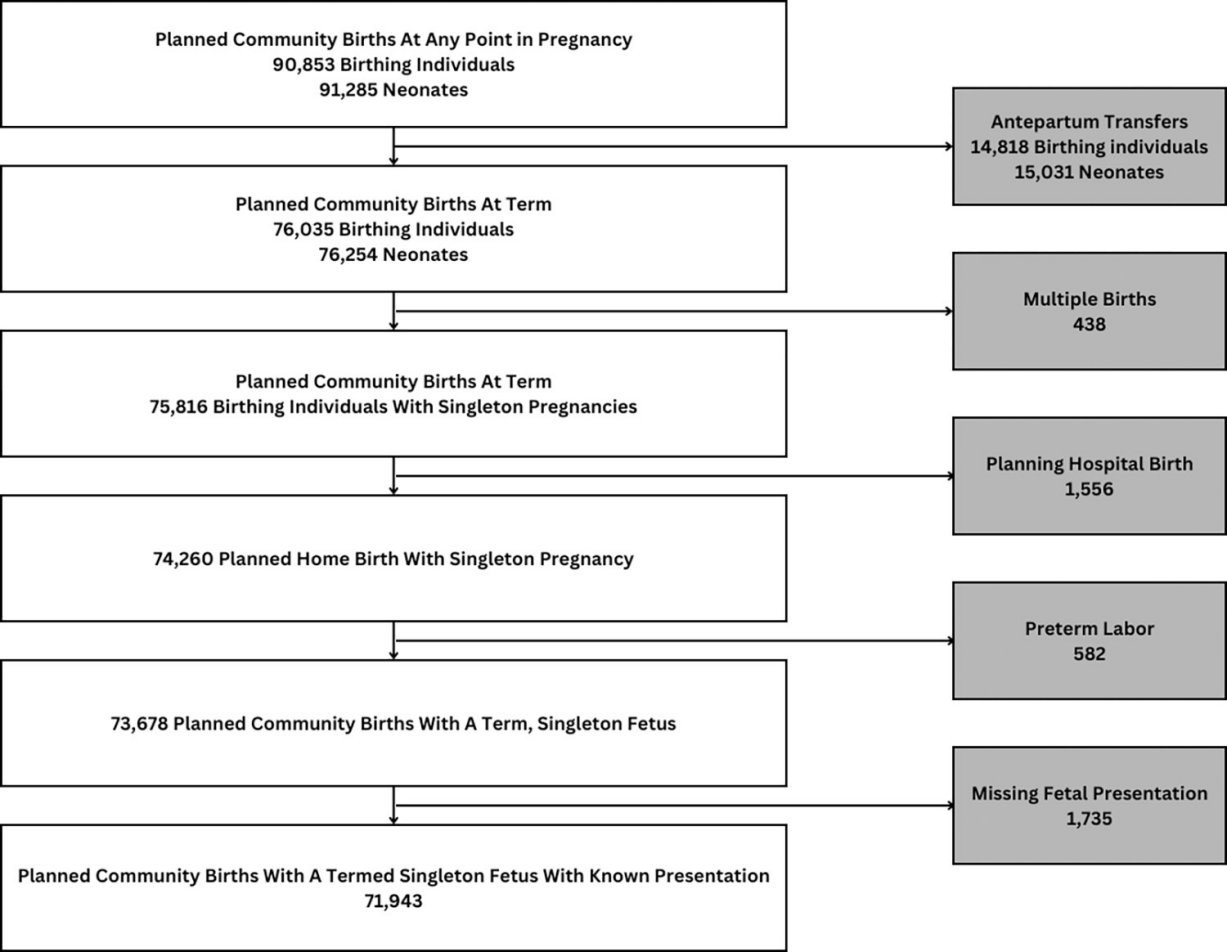
Study sample from MANA stats perinatal registry.

MANA data were accessed July 1, 2019. The study sample (N = 71,943) included all singleton, term births for individuals who planned community birth at the onset of labor and had a documented fetal presentation at birth (**Fig 1**). Pregnancies missing information on fetal presentation at birth were excluded, as were persons who changed their intended site of birth to a hospital setting prior to onset of labor. Both vaginal and cesarean births were included. The main exposure of interest was breech presentation at birth (n = 695) in comparison to cephalic presentation, subdivided by type of breech presentation as defined by the data set variable “breech presentation at birth” as frank, complete, footling, kneeling, or unknown. No formal definitions of breech types were provided to midwives entering data into the registry; those who were uncertain could contact MANA Stats support staff for assistance.

We explored associations between breech presentations at time of birth and multiple perinatal outcomes including durations of labor stages and membrane rupture. Labor stages were defined in the MANA Stats system as follows: first stage as the interval between frequent, intense contractions and onset of pushing; second stage as the start of active pushing efforts until birth of the neonate; and third stage as time from birth of the neonate until placental expulsion, as described in prior publications [42]. The management of impossible or improbable duration values are described in supplemental materials (**S1 Table**). Because this was a cohort of planned community births, intrapartum or postpartum transfer to hospital within six hours after birth was assessed, along with the reason(s) for transfer and urgency. Determination of indication(s) for transfer and associated urgency were based on assessment of the transferring midwife. We also analyzed maternal hospitalization in the first six weeks postpartum, including new admissions following community birth and postpartum readmissions. Finally, we evaluated adverse maternal outcomes, including severe (i.e., third- or fourth-degree) perineal laceration, retained placenta, and obstetric hemorrhage (defined as ≥1000 mL and/or diagnosed hemorrhage regardless of estimated blood loss) [43].

Neonatal outcomes included transfer to hospital in the first six hours of life (including indications and urgency), hospitalization (any) and/or NICU admission in the first six weeks of life (whether primary or readmission), umbilical cord prolapse, birth injury (defined as “skeletal fracture, peripheral nerve injury, and soft tissue or solid organ hemorrhage requiring intervention”), and intrapartum or neonatal death up to six weeks. Because term breech presentation is associated with congenital anomalies [44–46], we also assessed the presence of congenital anomalies (diagnosed antenatally or in the first six weeks of life) and explored deaths associated with anomalies separately. For every intrapartum or neonatal death, we explored free-text data entered by the community birth midwives describing the clinical course and circumstances surrounding care and provided brief case summaries.

Statistical analyses were performed using SPSS V 24.0.0.0 (IBM Corporation, Armonk, NY, USA) and R version 3.3.2 (R Foundation for Statistical Computing, Vienna, Austria). Initial analysis compared all types of breech presentation, collectively, to cephalic presentation. Analyses were then repeated to compare outcomes by presentation type. Medians and interquartile range are reported for labor durations and frequencies for all other outcomes. Because multivariable models were not possible due to low event counts for adverse outcomes, bivariable analyses were performed. We reported counts and proportions, including odds ratios (ORs) and confidence intervals (CI) for outcomes with five or more events in both comparison groups. Standard bivariable statistics were used to explore associations. We used unadjusted logistic regression models to calculate ORs and 95% CIs for categorical outcomes and the Kruskal-Wallis test to assess associations between breech presentation and labor duration, stratified by parity.

To contextualize our study sample, we compared the overall proportion of breech presentation to the expected proportion in the general US childbearing population based on vital statistics data (2016–2021) [47]. With the understanding that maternity care policies related to breech birth care may affect access to care and health outcomes [48], we also explored the two most frequent outcomes (cesarean and intrapartum transfer) for both cephalic and breech presentation stratified by covariables of planned site of community birth (i.e., home or birth center) and region of the country. Finally, since there is evidence that the level of integration of community birth providers into regional health systems affects maternal and neonatal birth outcomes [49], we explored associations state-level midwifery care integration scores (defined by Vedam et al., 2018) as an additional covariable in this analysis.

## Results

In this sample of 71,943 individuals, 1% (n = 695) gave birth to a term, singleton, breech neonate. Incidence of breech births in this low-risk sample of planned community births was, predictably, lower than the rate of 2.8% found the general US childbearing population (based on term, singleton births with known presentation from 2016–2021). As shown in **Table 2**, demographic characteristics of individuals in this sample who experienced breech birth were generally similar to those with a cephalic birth, except for increased likelihood of being nulliparous (48.7% breech, 32.6% cephalic) and not eligible for low-income public health insurance (19.5% breech, 23.2% cephalic). Of the 695 breech neonates in this sample, the majority presented frank breech at birth (57.0%, n = 396), followed by complete (19.3%, n = 134), footling (17.7%, n = 123), and kneeling (0.7%, n = 5) presentations. Type of breech presentation was unknown in 5.3% (n = 37) of births.

**Table 2:** Demographic characteristics of the study sample (stratified by fetal presentation).

Comparison variable	total n (%)	breech n (%)	cephalic n (%)	p-value (chi-square test)
total	71,943	695 (1.0%)	71,248 (99.0%)	
*Maternal characteristics*				
Age, *mean (SD)*	30.6 (5.0)	31.2 (5.0)	30.6 (5.0)	0.004^a^
Race identified as White	66,883 (93.2%)	660 (95.5%)	66,223 (93.2%)	0.02
Married or partnered	68,293 (94.9%)	667 (96.0%)	67,626 (94.9)	0.26
Level of education bachelor’s degree or higher	35,804 (50.3%)	349 (50.7%)	35,455 (50.3%)	0.85
Eligible for Medicaid (public health insurance) based on income	16,646 (23.2%)	135 (19.5%)	16,511 (23.2%)	0.02
Pre-gravid BMI <18.5 18.5–24.9 25–29.9 30–34.9 ≥ 35 Missing	2803 (3.9%) 42,753 (59.4%) 13,977 (19.4%) 5141 (7.1%) 2868 (4.0%) 4401 (6.1%)	30 (4.3%) 412 (59.3%) 136 (19.6%) 49 (7.1%) 25 (3.6%) 43 (6.2%)	2773 (3.9%) 42,341 (59.4%) 13,841 (19.4%) 5092 (7.1%) 2843 (4.0%) 4358 (6.1%)	0.99
Nulliparous	23,457 (32.6%)	338 (48.7%)	23,119 (32.5%)	<0.001
Parous, with: History of cesarean with prior vaginal birth	2072 (4.3%)^b^	14 (3.9%)^b^	2058 (4.3%)^b^	0.07
History of cesarean only	1756 (3.6%)^b^	21 (5.9%)^b^	1735 (3.6%)^b^	
*Pregnancy characteristics*				
Gestational age at birth, mean (SD)	281.5 (7.7)	279.5 (8.6)	281.5 (7.7)	<0.001
Post-dates gestation	2808 (3.9%)	19 (2.7%)	2789 (3.9%)	0.12
Planned place of birth home birth center	50,324 (69.9%) 21,619 (30.1%)	531 (76.4%) 164 (23.6%)	49,793 (69.9%) 21,455 (30.1%)	<0.001
*Provider characteristics*				
Primary provider credential Certified professional midwife (CPM) Certified nurse-midwife (CNM) Dually certified midwife (CPM/CNM) Other type of provider^c^	52,077 (72.4%) 8462 (11.8%) 2368 (3.3%) 9019 (12.5%)	524 (75.4%) 72 (10.4%) 19 (2.7%) 80 (11.5%)	51,553 (72.4%) 8390 (11.8%) 2349 (3.3%) 8939 (12.5%)	0.48

^a^ For maternal age, the p-value is from a t-test assuming equal variances

^b^ Denominator is multiparas

^c^ Other types of providers included student midwives under supervision, clinicians with other credentials (e.g., ND, DO, lay midwives), and unknown or missing provider credential information.

Notes: Data come from the Midwives Alliance of North America Statistics Project (MANA Stats), birth years 2012–2018. Comparison of demographic and pregnancy risk factor variables between births including a breech fetus, compared to births with a cephalic fetus. Sample was limited to singleton, not preterm, and not missing information on presentation.

Associations between breech presentation and maternal and neonatal outcomes are presented in **Table 3**, with reasons for transfer detailed and compared in **Table 4**. Nearly half (42.4%) of all breech neonates in planned community births were born via cesarean (versus 3.8% for cephalic), and, relatedly, more individuals with a breech fetus transferred from community birth settings to the hospital in the intrapartum period (OR 9.0, 95% CI 7.7–10.4). Midwives classified more breech intrapartum transfers as urgent (46% v. 17%, p < 0.001), with malpresentation/malposition (85%) being the most common reason for intrapartum transfer. Multiple indications for transfer were commonly cited. Other than cord prolapse and fetal malpresentation, all other reasons for transfer were more common among cephalic labors. After intrapartum transfer (n = 344), 50 breech neonates were born vaginally (14.5%, vs. 61.4% of cephalic intrapartum transfers) in hospital settings. Vaginal hospital births included 30 frank breech, 7 complete, 12 footling, and 1 unknown breech type.

**Table 3:** Maternal and neonatal outcomes, by fetal presentation.

Outcome	Cephalic n (%) N = 71,248	Breech n (%) N = 695	OR (95% CI)
**Maternal outcomes**			
Intrapartum transfer (any)	7030 (9.9%)	344 (49.5%)	9.0 (7.7–10.4)
Intrapartum transfer (urgent)	1171 (1.6%)	159 (22.9%)	17.7 (14.7–23.4)
Cesarean	2713 (3.8%)	294 (42.4%)	18.6 (15.9–21.7)
Postpartum transfer (any)^a^	1699 (2.6%)	22 (6.3%)	2.5 (1.6–3.8)
Postpartum transfer (urgent)	912 (1.4%)	13 (3.7%)	2.7 (1.5–4.6)
Severe perineal laceration^b^	948 (1.4%)	11 (2.8%)	2.0 (1.1–3.7)
Hemorrhage (any)	3836 (5.4%)	33 (4.7%)	0.88 (0.62–1.2)
Hemorrhage ≥1000 mL	1594 (2.4%)	9 (2.0%)	0.82 (0.42–1.6)
Hospitalization	1681 (2.4%)	21 (3.1%)	1.3 (0.86–2.1)
**Neonatal outcomes**			
Neonatal transfer (any)^a^	1126 (1.8%)	27 (7.7%)	4.7 (3.1–7.0)
Neonatal transfer (urgent)	727 (1.1%)	22 (6.3%)	5.8 (3.8–9.1)
Umbilical cord prolapse	50 (0.1%)	15 (2.2%)	32.2 (18.0–57.7)
Congenital anomaly (any)	627 (0.9%)	14 (2.0%)	2.3 (1.4–4.0)
Birth injury	212 (0.3%)	16 (2.3%)	7.9 (4.7–13.2)
Hospitalization (any)	2576 (3.6%)	30 (4.5%)	1.2 (0.86–1.8)
NICU admission	1868 (2.6%)	44 (6.6%)	2.6 (1.9–3.5)
Intrapartum or neonatal death (any)	122/71,248 (1.7/1000)	10/695 (14.4/1000)	8.5 (4.4–16.3)
Intrapartum or neonatal death (not attributed to congenital anomaly)	100/71,215 (1.4/1000)	8/693 (11.5/1000)	8.3 (4.0–17.1)

^a^ limited to those who completed community birth

^b^ limited to vaginal births; includes third- and fourth-degree lacerations

Notes: Odds Ratios are breech vs. cephalic, so OR > 1 means the outcome is more common in breech labors, and OR < 1 means outcome is less common in breech labors. All ORs are unadjusted because of small sample sizes.

**Table 4:** Reasons for intrapartum, postpartum, and neonatal transfer, by fetal presentation.

Reason for transfer^a^	Cephalic	Breech	Chi-square p-value^b^
***Intrapartum Transfer***	N = 7027	N = 344	
** Maternal indications**			
Arrest of labor/failure to progress, first stage of labor	2810 (**40.0%)**	27 (7.8%)	<0.001
Arrest of labor/failure to progress, second stage of labor	1154 (**16.4%)**	9 (2.6%)	<0.001
Prolonged labor	617 (**8.8%**)	4 (1.2%)	---
Prolonged rupture of membranes	1048 (**14.9%**)	18 (5.2%)	<0.001
Maternal dehydration	182 (**2.6%)**	0	---
Hypertensive disorders of pregnancy	213 (**3.0%**)	2 (0.6%)	---
Maternal exhaustion	1799 (**25.6%)**	8 (2.3%)	<0.001
Maternal request for additional pain relief	2387 (**34.0%**)	15 (4.4%)	<0.001
Signs or symptoms of infection	124 (**1.8%**)	0	---
Uterine rupture	6 (**0.1%**)	0	---
** Fetal indications**			
Umbilical cord prolapse	24 (0.3%)	7 (**2.0%)**	<0.001
Malposition or malpresentation	1273 (18.1%)	293 (**85.2%**)	<0.001
Light/thin meconium	408 (**5.8%**)	10 (2.9%)	<0.02
Heavy/thick meconium	501 (7.1%)	23 (6.7%)	0.83
Non-reassuring fetal heart tones	1101 (**15.7%**)	10 (2.9%)	<0.001
Placental abruption	70 (**1.0%)**	2 (0.6%)	---
Other^c^	506 (7.2%)	25 (7.3%)	0.92
***Postpartum Transfer***^d^ ***(< 6 hours after birth)***	N = 1707	N = 22	
Cervical or uterine prolapse	5 (0.3%)	0	---
Hemorrhage	677 (**39.7%)**	5 (22.7%)	0.13
Laceration repair	602 (**35.3%**)	6 (27.3%)	0.51
Hypertension	14 (0.8%)	0	---
Retained placenta	510 (**29.9%**)	6 (27.3%)	1.0
Signs/symptoms of infection	9 (0.5%)	0	---
Other reason^e^	241 (14.1%)	9 (**40.9%**)	0.002
***Neonatal Transfer (< 6 hours after birth)***	N = 1132	N = 27	
Birth trauma/injury	40 (3.5%)	5 (**18.5%**)	0.003
Suspected congenital anomaly	75 (6.6%)	0	---
Meconium aspiration syndrome	87 (7.7%)	0	---
Signs of prematurity	6 (0.5%)	1 (3.7%)	
Respiratory distress syndrome	687 (**60.7%)**	11 (40.7%)	0.05
Neonatal seizures	16 (1.4%)	0	---
Symptoms of infection	77 (36.8%)	1 (3.7%)	---
Other reason^f^	350 (30.9%)	15 (**55.6%)**	0.01

Notes

^a^ Multiple item selection permissible on data entry

^b^ P-values are suppressed unless there were at least 5 events in both groups.

^c^ Other reasons were reported as

Medical complications: abnormal vital signs, seizure, stroke, active herpes simplex infection, cardiac condition, excessive nausea, and vomiting

Obstetric complications: prolonged rupture of membranes, precipitous labor (unattended), hypertensive disorders of pregnancy, oligohydramnios, postterm gestation, cervical edema, urinary retention

Situational or environmental factors: poor weather conditions, independent maternal decision to transfer, state regulations, lack of availability of birth attendant

^d^ Postpartum and neonatal transfer include transfers within the first 6 hours after birth.

^e^ Other reasons were reported as

Medical complications: abnormal vital signs, postpartum psychosis, syncope

Obstetric complications: precipitous labor

Situational or environmental factors: maternal intuition (“didn’t feel right”), desire to remain with neonate requiring transfer

^f^ Other reasons were reported as

Neonatal complications: lethargy, cardiac arrythmia, unspecified (baby “didn’t look right”), protocol following resuscitation (not meeting criteria for respiratory distress syndrome)

Situational or environmental factors: precipitous and/or unattended birth, desire for neonate to remain with postpartum person requiring transfer

Maternal postpartum transfers were also more likely to be considered urgent in breech births (OR 2.7, 95% CI 1.5–4.6), even though prevailing maternal indications for transfer (including hemorrhage, laceration repair, and retained placenta) were more common in the cephalic group. Neither postpartum hemorrhage nor maternal hospitalization increased significantly with breech presentation compared to cephalic. There were insufficient events of operative births (i.e., forceps) (n = 4) or retained placenta (n = 7) for analysis.

**Fig 2:**
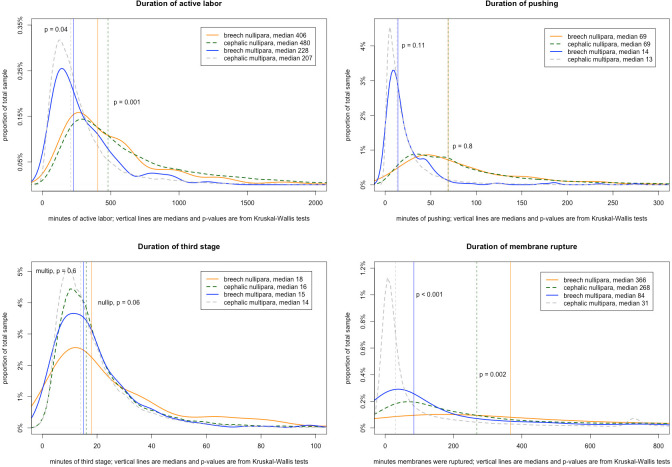
Durations of stages of labor and membrane rupture (comparing breech to cephalic, stratified by parity.

Distributions of labor duration variables are shown in **Fig 2**, stratified by presentation and parity. Median active labor for breech fetuses among nulliparas was shorter than cephalic fetuses (406 vs. 480 minutes), but the opposite was true for multiparous individuals (228 breech vs. 207 cephalic). There were no significant differences in duration of second or third stages based on fetal presentation, although breech labors were associated with significantly longer durations of membrane rupture for both nulliparas (median 336 minutes for breech vs. 268 cephalic) and multiparas (84 breech vs. 31 cephalic).

For neonates, breech presentation was associated with increased odds of neonatal transfer, NICU admission, and birth injury (OR 4.7, 95% CI 3.1–7.0; OR 2.6, 95% CI 1.9–3.5; and OR 7.9, 95% CI 4.7–13.2, respectively) (Table 3). There was no association between presentation at birth and neonatal hospitalization. Regarding indications for neonatal transfer (Table 4), breech neonates were more likely to transfer for birth injury (18.5% vs. 3.5%) and “other” (not listed) reasons (55.6% vs. 30.9%) and less likely to transfer for respiratory distress (40.7% vs. 60.7%). Breech births were also more likely to experience umbilical cord prolapse (2.2% v. 0.1%, OR 32.2, 95% CI 18.0–57.7).

There was also a substantive increase in odds of intrapartum or neonatal death for the breech fetus (OR 8.5, 95% CI 4.4–16.3). Although based on only ten perinatal deaths (five intrapartum and five neonatal), this association persisted even when deaths related to congenital anomalies were excluded (OR 8.3, 95% CI 4.0–17.1). Deaths (described in **S2 Table**) were attributed to congenital anomalies (n = 4), head entrapment (n = 3), cord prolapse (n = 2), and unknown causes (interoperative death, suspected placental abruption) (n = 1). Several intrapartum/neonatal deaths were complicated by late diagnosis of breech presentation and inefficient transfer of care including medical errors by emergency medical services (EMS), delays in hospital assessment and treatment, and conflicts with EMS or hospital staff. It is also worth noting that intrapartum/neonatal deaths included several instances of late onset of community-based care, with the midwives describing assuming responsibility for antepartum care only after hospital providers declined care for planned vaginal birth due to breech presentation in the absence of other risk factors.

Maternal and neonatal outcomes stratified by type of breech presentation are shown in **Table 5**. For many outcomes, the small sample size of breech births and correspondingly low event counts preclude firm conclusions; however, a few patterns do emerge from the limited data. Rates of intrapartum transfer and cesarean birth are similar across all breech types, and postpartum hemorrhage was less common with frank breech (3.3% frank vs. 6.0% complete, 7.0% footling/kneeling). Neonatal transfers, hospitalization, and NICU admissions were twice as common in footling/kneeling presentations. Umbilical cord prolapse was also significantly more common, occurring in 7.3% of footling/kneeling breech births (0.8% frank, 2.3% complete); however, perinatal death was half as likely (7.8/1000 footling/kneeling vs. 20/1000 frank, 22/1000 complete)—a finding that should be interpreted with caution given the low incidence of death (n = 1) in the footling/kneeling group.

**Table 5:** Maternal and neonatal outcomes, by type of breech presentation.

	Frank breech N = 396	Complete breech N = 134		Footling/kneeling breech N = 128	
Outcome	n (%)	n (%)	OR (95% CI)	n (%)	OR (95% CI)
**Maternal outcomes**					
Intrapartum transfer	189 (47.7%)	65 (48.5%)	1.03 (0.70–1.5)	65 (50.8%)	1.1 (0.76–1.7)
Cesarean	159 (40.3%)	58 (43.3%)	1.1 (0.76–1.7)	53 (41.4%)	1.0 (0.70–1.6)
Postpartum transfer^a^	11 (5.3%)	4 (5.8%)	---	5 (7.9%)	1.5 (0.52–4.6)
Severe perineal laceration	9 (2.3%)	1 (0.7%)	---	2 (1.6%)	---
Hemorrhage (any)	13 (3.3%)	8 (6.0%)	1.9 (0.76–4.6)	9 (7.0%)	2.2 (0.93–5.3)
Hemorrhage ≥1000 mL	4 (1.4%)	2 (2.4%)	---	2 (2.5%)	---
Hospitalization	10 (2.6%)	2 (1.5%)	---	6 (4.8%)	1.9 (0.68–5.3)
**Neonatal outcomes**					
Neonatal transfer^1^	15 (7.2%)	3 (4.4%)	---	8 (12.9%)	1.9 (0.77–4.7)
Umbilical cord prolapse	3 (0.8%)	3 (2.3%)	---	9 (7.3%)	---
Congenital anomaly, any	8 (2.0%)	3 (2.2%)	---	3 (2.3%)	---
Birth injury	8 (2.0%)	4 (3.0%)	---	4 (3.1%)	---
Hospitalization	14 (3.7%)	5 (3.8%)	1.0 (0.40–3.0)	9 (7.3%)	2.0 (0.86–4.8)
NICU admission	23 (6.1%)	8 (6.2%)	1.0 (0.44–2.3)	12 (9.6%)	1.6 (0.80–3.4)
Intrapartum or neonatal death (any)	6/396 (15.2/1000)	3/134 (22.4/1000)	---	1/128 (7.8/1000)	---
Intrapartum or neonatal death (not attributed to congenital anomaly)	4/394 (10.1/1000)	3/134 (22.4/1000)	---	1/128 (7.8/1000)	---

^a^ limited to those who completed community birth: 64,176 cephalic, 208 frank, 68 complete, 62 footling or kneeling presentations

Notes

Data are from planned community births in the USA, 2012–2018, limited to singleton term labors for which fetal presentation at birth was identified. Odds ratios use frank breech as the reference group (i.e., complete vs. frank; footling/kneeling vs. frank). Breech presentations of unknown type (N = 37) were excluded from this analysis.

Odds ratios have been suppressed for any category for which there were <5 events in either the numerator or denominator.

Finally, analysis of contextual variables (S3 **Table**) found higher rates of cesarean and intrapartum transfer for breech labors in the New England region (OR 17.6, 95% CI 7.6–40.9 and OR 47.2, 95% CI 20.1–110.7, respectively) compared to other regions of the country. There were no substantive differences in outcomes based on planned site of community birth (i.e., home or birth center) or level of integration of community birth midwifery services into the healthcare system, as defined by Vedam et al.[49]

## Discussion

Among this sample of planned community births, breech presentation was associated with high rates of intrapartum transfer and cesarean birth (OR 9.0 and 18.6, respectively) and no increased risk of maternal hospitalization or postpartum hemorrhage. Associations with nearly all assessed adverse neonatal outcomes were increased in breech births, including transfer, NICU admission, and birth injury. Umbilical cord prolapse occurred in 2.2% of breech births (OR 32.2, 95% CI 18.0–57.7). There was a high rate of intrapartum and neonatal death (14.4/1000, OR 8.5, 95% CI 4.4–16.3), which persisted even after excluding congenital anomalies.

All types of breech presentation carry additional risk for adverse neonatal outcomes. Although sample sizes precluded meaningful analysis of perinatal outcomes associated with type of breech presentation, our findings support existing research that increased incidence of umbilical cord prolapse in footling/kneeling breech presentations may not be associated with increased risk of severe complications [50], though this result should be interpreted with caution. Labor duration was not affected by type of breech presentation, as consistent with prior findings [51]. Although there was some regional variation in rates of maternal transfer and cesarean, there were no substantive differences in outcomes based on parity, planned site of birth, or level of care integration of community-based midwifery services.

Due to logistical and ethical concerns about randomizing individuals to site or mode of birth [10, 52, 53], assessment of outcomes associated with breech presentation relies primarily on observational evidence. This descriptive analysis is useful for guiding decision-making for breech labor and birth. The size and scope of this dataset are a strength of this study, with a large sample of individuals across community birth settings throughout the United States and high rates of participation in data collection from community midwives (>95%) [40]. Prospective enrollment in pregnancy ensured that all birth outcomes were included, thereby minimizing selection bias and potential underreporting of adverse outcomes [40]. Additionally, this dataset includes vaginal breech births and footling/kneeling presentations, which are often excluded from research.

Despite these strengths, there are also several limitations to the research based on this dataset. First, because participation in data collection is voluntary, outcomes may differ between providers who participate in data collection and those who do not. Second, as with any dataset, research findings are limited by the existing variables and their definitions. For example, because community birth providers avoid frequent or unnecessary cervical examinations, the dataset defined onset of second stage by initiation of pushing (rather than with onset of full cervical dilation as it is commonly defined). Although these definitions are used elsewhere in the literature [42], these findings may not correlate exactly to other studies exploring labor durations. Similarly, the lack of variables regarding comprehensive clinical and environmental factors prohibited investigation of predictive factors associated with breech birth outcomes. For example, we could not distinguish between planned and unplanned breech births, assess relationships with external cephalic version, determine when breech presentation was identified or whether a skilled breech attendant was present, or correlate outcomes with regulatory scope of practice restrictions, such as state regulations that limit community birth providers’ care for breech labors.

One additional limitation of this study is the possibility that not all presentation types were classified accurately. In community birth settings, there is rarely access ultrasound technology to confirm presentation, and evidence has demonstrated poor reliability in determining presentation by physical examination alone [54]. Due to constraints of existing breech nomenclature, there was also potential for unreliable classifications of presentation variants (such as when the hips and knees are incompletely flexed or feet are located alongside or just below the buttocks) or those that changed during labor (such as a complete breech fetus who extends a leg). Finally, because community birth care utilizes low levels of intervention, findings from breech community birth may not be generalizable to high-resource hospital settings [14].

### Interpretation and implications

Findings from this study reinforce existing evidence of increased risk of adverse neonatal outcomes in breech community birth [2, 55, 56]. Although many emergent interventions and technologies are not readily accessible in community births, the physiologic approach exemplified in these settings is widely considered by expert breech clinicians to be optimal for perinatal outcomes [57, 58]. However, even physiologic management in a low-risk population does not appear to circumvent risks to the breech neonate.

This research has implications for clinical practice, health care policy, and future research. Pregnant people should be counselled about the increased risk of adverse neonatal outcomes for breech fetuses in planned community births. These risks should be considered in context of the risks and benefits associated with sites and modes of birth, including risks to future pregnancies and individuals’ unique needs, preferences, values, and risk tolerance [13, 17]. Care providers in all settings should take steps to identify breech presentation at term and provide evidence-based information about breech birth outcomes to ensure informed choice. Skills in breech assessment and management should be incorporated into midwifery and obstetric training to optimize outcomes. Recognizing that breech community births will inevitably occur, both accidentally and intentionally, community and hospital birth providers should develop guidelines to identify and manage complications and provide timely and efficient transfer when needed [59].

Community birth is not well integrated into the health care system throughout the United States [49, 60], and this lack of coordination of care across birth settings was evident in several intrapartum and neonatal deaths in this sample. In addition, it was noted in a few cases that individuals were late to community-based care after they were declined care for planned hospital vaginal birth due to breech presentation in the absence of other risk factors. Community birth in the presence of high-risk conditions often indicates a failure of the medical system to meet patient’s needs for less interventive care and autonomy in decision-making [27, 61–64]. Restrictive policies preventing hospital providers from offering care for planned vaginal breech birth to appropriate candidates should be eliminated as they impede patient autonomy and access to care and inadvertently push more medically complex births into community settings [24, 26, 57, 59, 65, 66].

In prior published analyses using this data set, members of this research team recommended that, due to increased risk of adverse outcomes in community birth, breech presentations were better managed in birth settings with immediate access to hospital staff and facilities [2]. However, despite US recommendations supporting care for planned vaginal breech birth for appropriately screened candidates in hospitals [17, 22], access to vaginal breech birth and skilled breech providers in hospitals remains limited [25, 26]. Findings from this study, along with the recent increase in US breech community births, reinforce consensus recommendations that US hospitals have a “clear and urgent responsibility” [25] to increase access to care for planned vaginal breech given the increased risk of adverse perinatal outcomes associated with breech community birth compared to cephalic presentations. Policies and medicolegal reforms that incorporate best available evidence and center the birthing person and their rights to autonomy are necessary to improve maternal and neonatal outcomes and support informed choice for breech pregnancy and birth.

Breech presentation in all birth settings is associated with increased risk of adverse outcomes compared to cephalic presentation, and further research is needed to explore maternal and neonatal outcomes in matched cohorts of breech births in different settings with skilled breech providers. There is a need for development and adoption of a consistent and well-defined breech nomenclature to minimize ambiguity between presentation types and facilitate evidence synthesis. Future studies should explore outcomes based on type of breech presentation using this standardized nomenclature and report outcomes according to a standardized core outcome set (e.g., Breech-COS, currently in development) [67]. Research on breech labor outcomes is needed to guide decision-making, given that comparisons of prelabor cesarean to planned vaginal birth are not generalizable to laboring persons facing either emergent cesarean or unplanned vaginal breech birth. Researchers should assess the proportion of breech presentations correlated with underlying conditions (i.e., fetal growth restriction, congenital anomalies, oligohydramnios, placenta previa, maternal gestational diabetes mellitus or hypertensive disorders, uterine malformation, or history of cesarean) [45, 68] and investigate how these conditions affect morbidity and mortality, regardless of mode or site of birth. Finally, research is needed to explore the barriers and facilitators of breech birth care in the United States to guide recommendations to improve access to quality care [26].

## Conclusion

In planned community births, all types of breech presentation pose substantial risk of adverse outcomes, including high rates of intrapartum and neonatal death. This research provides evidence about breech labor in community birth settings and adverse maternal and neonatal outcomes associated with breech birth to inform decision-making. There is a need for increased training and research on vaginal breech birth. Reforms are needed to ensure accessible, high-quality care for planned vaginal breech birth in US hospitals.

## Supporting information

S1 Appendix Strengthening the reporting of observational studies in epidemiology (STROBE) statement. (DOCX)

S1 Table Management of impossible and improbable data for labor duration variables. (DOCX)

S2 Table Details for the 10 breech intrapartum/neonatal deaths. (DOCX)

S3 Table Cesarean and intrapartum transfer, by fetal presentation (stratified by region, level of care integration, and planned site of birth). (DOCX)
