# Supplementary material for: Maternal and neonatal outcomes associated with breech presentation in planned community (home and birth center) births in the United States: A prospective observational cohort study
Source: PLoS One. 2024 Jul 22;19(7):e0305587. doi: 10.1371/journal.pone.0305587 (PMC11262641; doi:10.1371/journal.pone.0305587)
Supplement: S1 Table — (DOCX) [file pone.0305587.s002.docx]

**S1 Table. Management of impossible and improbable data for labor duration variables**

| **Stage of Labor** | **Description of data management** | **Adjustments** | |
| --- | --- | --- | --- |
|  |  | **Nulliparas** | **Multiparas** |
|  |  | **n=23,457 (33%)** | **n=48,478 (67%)** |
| Active labor | Any that were >4320 minutes (3 days), we assumed were data entry errors, so were set to the sample medians, separately for primiparas and multiparas | 29 cases with duration of active labor > 4320 minutes, set to nulliparous sample median of 480 minutes; this changed the overall nulliparous sample mean from 654 minutes to 648 | 5 cases with duration > 4320 minutes, set to multiparous sample median of 207 minutes; this changed the overall multiparous sample mean from 288 to 287 |
| Second stage (pushing) | Any that were < 0 minutes or > 720 minutes (12 hours), assumed to be data entry errors, set to sample medians | 336 were > 720 and 8 were < 0   - Excluding the ones < 0 (some of which were pushing for negative 10 years—clearly year data entry errors in the dates), the initial sample wide median was 69 and the mean was 110. - After setting the 344 to 69 instead, the new mean was 96 minutes. | 222 were > 720 and none were < 0   - The initial median was 13 minutes, and the mean was 28 minutes. - After setting the 222 to 13, the new mean was 24. |
| Third stage | Any that were > 720 minutes (12 hours), assumed to be data entry errors and set to sample medians. Twelve hours was used here, even though third stage is expected to be much shorter than pushing stage, to allow for very long third stages in case of retained placentas, which require postpartum transfer to a hospital if this occurs in community settings | 215 were > 720 and were changed to the sample median of 16; this changed the overall mean from 30 to 22 | 213 were > 720 and were changed to the sample median of 14; this changed the overall mean from 22 to 19 |
| Membrane rupture | Any that were >4320 minutes (3 days) or < 0 minutes were assumed to be data entry errors and set to the sample medians | 270 needed to be fixed (29 were < 0)—set to the sample median of 269; this changed the mean (calculated without the < 0 records) from 1003 minutes to 584 | 140 needed to be fixed (23 were < 0)—set to sample median of 31; this changed the mean (calculated without the < 0 records) from 301 to 226 |
