# Supplementary material for: Maternal and neonatal outcomes associated with breech presentation in planned community (home and birth center) births in the United States: A prospective observational cohort study
Source: PLoS One. 2024 Jul 22;19(7):e0305587. doi: 10.1371/journal.pone.0305587 (PMC11262641; doi:10.1371/journal.pone.0305587)
Supplement: S2 Table — (DOCX) [file pone.0305587.s003.docx]

**S2 Table. Details for the 10 breech intrapartum/neonatal deaths**

| **Timing of death** | **Description of each case** |
| --- | --- |
| Intrapartum | 1. Unanticipated breech presentation in person with high BMI. SROM in early labor, patient called the midwife a few hours later reporting cord prolapse. EMS was called, nearest hospital was an hour away. Neonate died of hypoxia. 2. Great grand multipara. Unclear if known breech presentation, late transfer to community birth care. Midwife’s arrival was delayed due to weather, was called *en route* and informed feet were protruding from the vagina. EMS was called. Midwife arrived and diagnosed and managed head entrapment. Apgar 0, resuscitation unsuccessful. EMS arrived and transported the deceased neonate. 3. SGA fetus born with multiple nuchal cord loops and evidence of congenital malformations. Unclear if known breech presentation. Fetal anatomy ultrasound and prenatal testing showed no evidence of abnormalities. Cause of death as per autopsy was strangulation, possible abruption. 4. Known breech presentation in an individual with history of prior IUFD. Midwife diagnosed a cord prolapse in active labor. EMS was called and transported laboring patient, while the midwife elevated the presenting part off the cord. Following transfer, cesarean was performed under general anesthesia. Neonatal resuscitation was attempted but unsuccessful. 5. Unanticipated breech presentation, diagnosed in active labor. Vaginal bleeding present. Intrapartum transfer to hospital by personal vehicle. Fetal heart tones were reassuring following hospital transfer and present at start of nonemergent cesarean, which was delayed following admission. Interoperative fetal death. No documented cause of death, although the midwife was informed by nursing staff that there had been clinical evidence of placental abruption. |
| Neonatal | 1. DOL 0, unplanned breech birth in community setting. EMS called promptly, resuscitation immediately after birth unsuccessful. Neonate died during transport. EMS lacked necessary skills and equipment for resuscitation. Autopsy revealed cardiac anomaly and renal agenesis. 2. DOL 3, unplanned breech birth in community setting. EMS called promptly, resuscitation immediately after birth unsuccessful, improper intubation by EMS. Interpersonal conflict on hospital admission. Neonate admitted to NICU, life support discontinued after 3 days. Chromosomal anomalies (non-specified) present. 3. DOL 4, planned home breech birth. Head entrapment diagnosed and managed, resuscitation prior to EMS arrival. NICU admission. Cause of death not specified. 4. DOL 0, planned home breech birth of fetus with multiple known congenital anomalies incompatible with life, DNR in place. 5. DOL 0, planned home breech birth. Head entrapment diagnosed and managed, resuscitation immediately after birth unsuccessful. Neonate died prior to EMS arrival, post-mortem transfer to hospital with conflict. |

Abbreviations used: BMI, body mass index; DNR, do not resuscitate order; DOL, day(s) of life; EMS, emergency medical services; IUFD, intrauterine fetal demise; midwife, midwife; NICU, neonatal intensive care; OB, obstetrician; SGA, small for gestational age; SROM, spontaneous rupture of membranes; VBAC, vaginal birth after cesarean; WNL, within normal limits
