# Supplementary material for: Maternal and neonatal outcomes associated with breech presentation in planned community (home and birth center) births in the United States: A prospective observational cohort study
Source: PLoS One. 2024 Jul 22;19(7):e0305587. doi: 10.1371/journal.pone.0305587 (PMC11262641; doi:10.1371/journal.pone.0305587)
Supplement: S3 Table — (DOCX) [file pone.0305587.s004.docx]

|  |  | **Intrapartum transfer** | | | **Cesarean** | | | **Transfers who had a cesarean** | |
| --- | --- | --- | --- | --- | --- | --- | --- | --- | --- |
|  | **Overall % breech** | **n (%) cephalic** | **n (%) breech** | **OR**  **(95% CI)** | **n (%) cephalic** | **n (%) breech** | **OR (95% CI)** | **n (%) cephalic** | **n (%)**  **breech** |
| **Region of the country**^a^ | | | | | | | | | |
| Region 1:  New England | 26/2794  0.9% | 316  (11.3%) | 18  (69.2%) | 17.6  (7.6 - 40.9) | 127  (4.5%) | 18  (69.2%) | 47.2  (20.1 - 110.7) | 127/316  (40.2%) | 18/18  (100%) |
| Region 2: Midatlantic | 85/7343  1.1% | 581  (7.9%) | 34  (40.0%) | 7.6  (5.0 - 12.1) | 258  (3.5%) | 29  (34.1%) | 14.2  (8.9 - 22.6) | 258/581  (44.4%) | 29/34  (85.3%) |
| Region 3:  Southeast | 76/6864  1.1% | 583  (8.5%) | 38  (50.0%) | 10.8  (6.8 - 17.0) | 223  (3.2%) | 27  (35.5%) | 16.4  (10.1 - 26.7) | 223/583  (38.3%) | 27/38  (71.1%) |
| Region 4: Midwest | 125/10,626  1.2% | 864  (8.1%) | 63  (50.4%) | 11.5  (8.0 - 16.4) | 304  (2.9%) | 50  (40.3%) | 22.9  (15.7 - 33.4) | 304/864  (35.2%) | 50/63  (79.4%) |
| Region 5: Southwest | 144/13,185  1.1% | 1240  (9.4%) | 56  (38.9%) | 6.1  (4.4 - 8.6) | 493  (3.7%) | 52  (36.1%) | 14.5  (10.2 - 20.7) | 493/1240  (39.8%) | 52/56  (92.9%) |
| Region 6:  West | 232/29,534  0.8% | 3331  (11.3%) | 131  (56.5%) | 10.2  (7.9 - 13.3) | 1259  (4.3%) | 114  (49.1%) | 21.7  (16.7 - 28.2) | 1259/3331  (37.8%) | 114/131  (87.0%) |
| **Level of integration of care of community birth midwifery services into the healthcare system**^b^ | | | | | | | | | |
| Low integration^c,d^ | 161/13,499  1.2% | 1198  (9.0%) | 86  (53.4%) | 11.6  (8.5 - 15.9) | 467  (3.5%) | 73  (45.6%) | 23.1  (16.7 - 32.0) | 467/1198  (39.0%) | 73/86  (84.9%) |
| High integration^c,e^ | 527/57,535  0.9% | 5717  (10.0%) | 254  (48.2%) | 8.3  (7.0 - 9.9) | 2197  (3.9%) | 217  (41.2%) | 17.5  (14.6 - 20.9) | 2197/5717  (38.4%) | 217/254  (85.4%) |
| **Planned place of birth** | | | | | | | | | |
| Home | 531/79,793  1.1% | 4648  (9.3%) | 246  (46.3%) | 8.4  (7.0 - 10.0) | 1813  (3.6%) | 209  (39.4%) | 17.2  (14.4 - 20.6) | 1813/4648  (39.0%) | 209/246  (85.0%) |
| Birth center | 164/21,455  0.8% | 2382  (11.1%) | 98  (59.8%) | 11.9  (8.7 - 16.3) | 900  (4.2%) | 85  (51.8%) | 24.6  (18.0 - 33.6) | 900/2382  (37.8%) | 85/98  (86.7%) |

^______^

**Notes:**

^a^ States included in each region are as follows: Region 1: CT, MA, ME, NH, RI, VT; Region 2: DC, DE, NJ, NY, MD, PA; Region 3: AL, AR, FL, GA, LA, MS, NC, KY, SC, TN, VA, WV; Region 4: IA, IL, IN, KS, MI, MN, MO, ND, NE, OH, SD, WI; Region 5: AZ, CO, ID, MT, NM, NV, OK, TX, UT, WY; Region 6: AK, CA, HI, OR, WA.

^b^ Level of care integration as determined by Vedam S, Stoll K, MacDorman M, Declercq E, Cramer R, Cheyney M, et al. Mapping integration of midwives across the United States: Impact on access, equity, and outcomes. PLoS One. 2018;13(2):e0192523.

^c^ Low and high are defined as below or above the median of 37, so low: 0-37 and high 38-100.

^d^ Low integration group, in increasing order of score, include: NC, AL, SD, OH, KS, MS, NE, OK, HI, IL, IA, KY, PA, CT, LA, DC, NV, GA, ND, MA, AR, WV, MI, SC, IN, ME.

^e^ High integration, in increasing order, includes: MD, MO, CO, TN, VA, DE, FL, TX, WI, AK, CA, WY, VT, MN, NH, UT, ID, MT, AZ, RI, NY, NJ, OR, NM, WA.
